# Changes in Sitting Time and Sitting Fragmentation after a Workplace Sedentary Behaviour Intervention

**DOI:** 10.3390/ijerph15061148

**Published:** 2018-06-01

**Authors:** Jasmin Hutchinson, Samuel Headley, Tracey Matthews, Greg Spicer, Kristen Dempsey, Sarah Wooley, Xanne Janssen

**Affiliations:** 1 Department of Exercise Science and Sport Studies, Springfield College, 263 Alden Street, Springfield, MA 01109, USA; sheadley@springfieldcollege.edu (S.H.); gspicer@springfieldcollege.edu (G.S.); swooley@springfieldcollege.edu (S.W.); 2 School of Health, Physical Education and Recreation, Springfield College, 263 Alden Street, Springfield, MA 01109, USA; tmatthews@springfieldcollege.edu; 3 Cardiac Rehab/Non-Invasive Cardiology, Newton-Wellesley Hospital, 2014 Washington Street, Newton, MA 02462, USA; dempseykristen1214@gmail.com; 4 School of Psychological Science and Health, University of Strathclyde, 16 Richmond Street, Glasgow G1 1XQ, UK; xanne.janssen@strath.ac.uk

**Keywords:** sitting time, occupational, sedentary fragmentation, objective measurement

## Abstract

Prolonged sedentary behaviour (SB) has shown to be detrimental to health. Nevertheless, population levels of SB are high and interventions to decrease SB are needed. This study aimed to explore the effect of a personalized intervention aimed at reducing SB and increasing breaks in SB among college employees. A pre-experimental study design was used. Participants (*n* = 36) were recruited at a college in Massachusetts, USA. SB was measured over 7 consecutive days using an activPAL3 accelerometer. Following baseline measures, all participants received a personalized SB consultation which focused on limiting bouts of SB >30 min, participants also received weekly follow-up e-mails. Post-intervention measures were taken after 16 weeks. Primary outcome variables were sedentary minutes/day and SB bouts >30 min. Differences between baseline and follow-up were analyzed using paired t-tests. The intervention did not change daily sedentary time (−0.48%; *p* > 0.05). The number of sedentary bouts >30 min decreased significantly by 0.52 bouts/day (*p* = 0.010). In this study, a personalized SB intervention was successful in reducing number of bouts >30 min of SB. However, daily sedentary time did not reduce significantly. These results indicate that personalized, consultation-based interventions may be effective if focused on a specific component of SB.

## 1. Introduction

Sedentary behavior (SB), defined as “any waking behavior characterized by an energy expenditure ≤ 1.5 metabolic equivalents (METS) while in a sitting or reclining posture” [1] is an important risk factor for poor health. Recent systematic reviews have linked high levels of SB to many negative health outcomes such as cardiovascular disease, type 2 diabetes, certain cancers, and all-cause mortality, independent of physical activity [2,3,4,5,6]. Nevertheless, sedentary behaviour is on the rise, particularly in developed countries. Adults in the United States [7], Norway [8] and Sweden [9] spend approximately two thirds of the waking day sedentary. Causes of SB in both developed and developing countries include reduced frequency of physical activity, increased sedentary leisure pursuits at home and increased amounts of seated technical work or desk-based office work [10]. Data from a range of industrialised countries indicate that increased sedentariness at work is an international phenomenon [11]. For example, Australian office workers reportedly spend 82% of their working day seated [12]. The workplace has been recognized as an important setting for the implementation of strategies to promote physical activity and reduce SB [13]. The workplace also presents opportunities to promote active commuting, and to build upon the rapidly growing practice of mobile health (or mHealth) to harness the potential of technology to help improve the health and wellbeing of all individuals [14]. Workplace SB interventions can be an effective way to reduce sedentary time and increase breaks in sedentary behaviour [15]. Most of these interventions have targeted the environment, for example implementing sit-stand desks or active workstations [16,17], and while these interventions can be effective, the question is whether these interventions are affordable and feasible to implement on a larger scale. A more affordable strategy, which is able to reach many people at once, is the use of digital applications to prompt employees to stand up at regular intervals [18]. However these interventions often lack an informative component, thereby failing to increase participant’s knowledge as to why they should be reducing SB. There is also a need to assess longer-term effects of such interventions. Another cost-effective workplace intervention recommended by the World Health Organization is behavioural counseling [19].

Previous research in physical activity has shown that including concepts such as personalized goal-setting and information prompts are important concepts to implement in behaviour change interventions [20]. In addition, individualized consultation approaches have shown to be an effective way to target these concepts and result in successful behaviour change in physical activity and dietary studies [20,21]. However, relatively few studies have implemented a personalized consultation based upon current patterns of behaviour. An exception is a small pilot study by Fitzsimons et al. in which community dwelling older adults (mean age = 68 ± 6 years) received a personalized SB consultation incorporating feedback from an activPAL activity monitor. Objectively measured daily time spent sitting/lying was reduced by 2.2% or 25 min per 24 h over 2 weeks. The intervention also significantly increased total time spent stepping by 13 min/day [22]. To the best of our knowledge, no study has taken a personalized approach to SB behavioural counseling in the workplace. Therefore, this study aimed to explore the feasibility and effectiveness of an individually tailored behavioural consultation aimed at reducing SB and increasing breaks in sedentary time in college workers.

## 2. Materials and Methods

### 2.1. Study Design

This study aimed to test the feasibility and pilot the effectiveness of a personalized, consultation based SB intervention in the workplace. The study was conducted as a pre-experimental (one group pretest–posttest) study design. Participants were enrolled in the study between September–December 2016 and follow-up data were collected in February–May 2017. The study was reviewed and approved by the Institutional Review Board of the College and data were only collected on individuals who gave their informed consent to participate.

### 2.2. Participants

**Figure 1:**
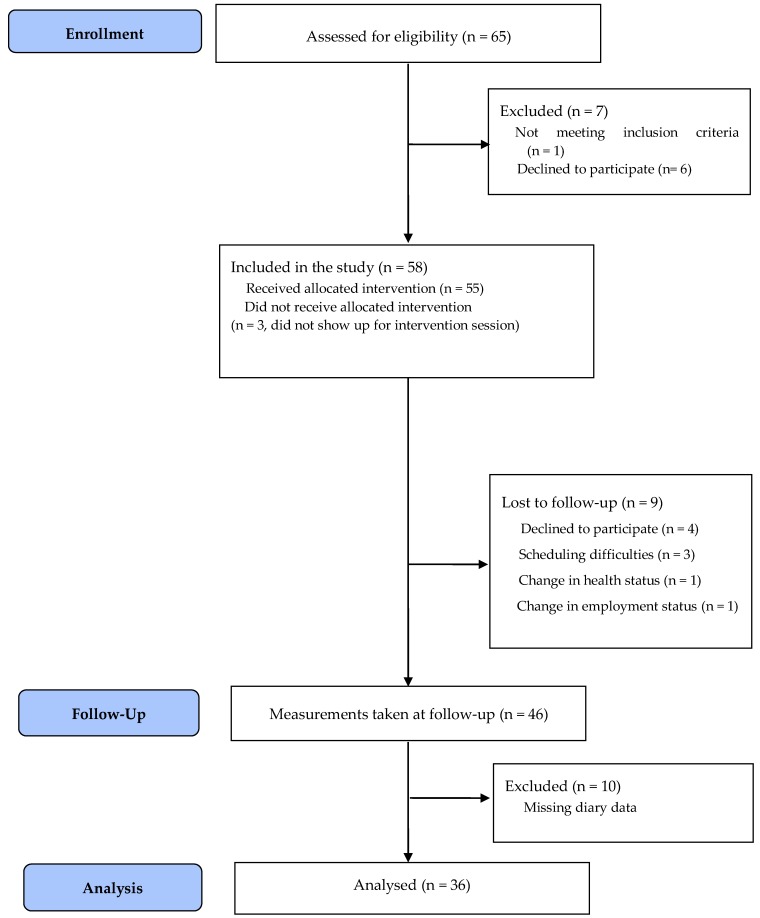
CONSORT flow diagram showing the flow of participants through each stage of the study.

Thirty-six participants (7 men, 29 women; mean age, 51.1 ± 11.1 years; mean BMI, 29.2 ± 7.6 kg/m^2^) were recruited from Springfield College (Springfield, MA, USA). All College employees received a recruitment email about the study at the beginning of the fall semester (i.e., September 2016). To be eligible to participate, participants had to be at least 18 years old and classified as full-time employees at the institution. Figure 1 shows the number of participants who were screened for eligibility, received the intervention, attended follow-up testing, and were included in the final analysis.

### 2.3. Procedures

During the first visit, participants provided informed consent and completed a brief demographic questionnaire. Height and weight were taken following standardized methods and participants were fitted with an activPAL3 monitor [23]. The monitor was attached directly to the midline anterior aspect of the participants’ right thigh, mid-way between the hip and the knee in the correct orientation as outlined by the manufacturer’s instructions. A nitral sleeve was used for waterproofing, and the monitor was secured to the thigh using Tegaderm dressing. Participants wore the activPal3 monitor continuously 24-h per day for seven consecutive days, after which the device was returned to the laboratory. During the 7-day wear period, participants were asked to record in a diary their bed (i.e., “lights out”) and wake times as well as the times they were at work each day.

A second visit was scheduled for one week after the activPAL3 monitor was returned, this was done in order to allow time for data processing. During this visit, participants met in small groups of 2–5 to participate in a personalized SB consultation, as outlined below. A third and final visit was scheduled 16-weeks following the behavioural intervention, at which time participants were asked to wear the activPAL3 for another seven consecutive days.

### 2.4. Intervention

Phase One: The behaviour change intervention consisted of one 45-min face-to-face consultation session conducted by a member of the research team, and a series of weekly follow-up emails delivered over the ensuing 16 weeks. The theoretical underpinning of the intervention was Lewin’s force field theory. Lewin (1947) put forward the idea, that behavioural status quo represents an equilibrium between forces favoring change (i.e., driving forces) and barriers to change (i.e., restraining forces) [24]. For a goal-directed activity to be successfully implemented, the magnitude of the driving force needs to match the magnitude of the restraining force [25].

The behavioural intervention sought to increase driving forces for change and reduce restraining forces. The behavioural intervention was delivered in five stages. The first stage focused on increasing participant’s knowledge of SB and the health effects of SB. During the second stage participants identified specific driving forces toward decreasing SB (the “why” of behaviour change). Participants also reviewed and reflected upon their current SB patterns based on their personal activPAL data. Using a printout of the 7-day report participants were able to identify the most sedentary periods of their work day and map these time periods to specific work tasks and behaviours. Stage 3 focused on finding feasible ways to reduce SB throughout the working day; this was achieved through brainstorming and facilitated group discussion. In the fourth stage potential barriers to change (i.e., restraining factors) were identified and solutions were sought, again through a process of self-reflection and group discussion. In the final stage additional behavioural strategies were offered (if not self-identified by the group) and participants created feasible goals to reduce their SB at work, specifically to break up bouts of SB greater than 30 min. Table 1 details the intervention in terms of specific behaviour change techniques, to allow for coding using the Behaviour Change Technique Taxonomy [26].

Phase 2: Following the behavioural consultation the intervention group received weekly e-mail prompts/reminders to break up prolonged bouts of sitting at work. Emails were sent every Monday morning during work hours. Content of the emails varied between short simple messages, graphical illustrations, information sharing (e.g., links to relevant content, or “did you know…?” statements) and specific tips on how to reduce or interrupt workplace sitting. Emails were designed to target both affective and cognitive attitudes [27] toward SB and included a combination of both gain-framed and loss-framed message content [28]. Full content of the weekly emails can be requested from the first author.

### 2.5. Outcome Measures and Statistical Analysis

Sedentary behaviour was measured using an activPAL3 accelerometer. The activPAL3 classifies a person’s behaviour into sitting/lying, standing and stepping and has been shown to be a valid and reliable measure of SB in adults [29]. Data were analysed using the event files from the activPAL3 and a personalized macro (available upon request from Xanne Janssen). Primary outcome measures were the average percentage of time spent sitting or lying per day/working day and the average number of bouts per day/working day lasting more than 30 min. Secondary outcomes were the average percentage of time spent standing and stepping per day/working day, sitting to upright transitions (lasting more than 1 min) and the average number of SB bouts lasting 10–19.99 min, 20–29.99 min and >30 min per day/working day.

Paired *t*-tests were used to compare time spent sitting/lying, standing and stepping, number of sitting to upright transitions and number of bouts lasting 10–10.99 min, 20–29.99 min and >30min between baseline and follow-up. A covariate model was considered, with BMI as a covariate, but non-significant correlations indicated that the basic assumption that the covariate is related to the dependent variables [30] was not met. Sedentary, standing and stepping time was expressed in percentages in the analysis to control for differences in waking time. A *p*-value of <0.05 was considered statistically significant. All statistical analyses were conducted in SPSS version 25 (SPSS, Inc., Chicago, IL, USA).

## 3. Results

Thirty-six participants provided informed consent and took part in the intervention. All participants provided at least 5 days of valid activPAL data for both baseline and follow-up measures. At baseline, participants spent an average of 9.4 h per day sedentary with no significant difference between females and males during the waking day (*p* = 0.563). See Table 2.

### 3.1. Whole Day Sedentary Behaviour

Intervention results are displayed in Table 3. Briefly, participants spent an average of 59.1% (SD 8.3) of their waking day sedentary at baseline (9.4 ± 1.5 h/day) and this decreased to 58.6% (SD 11.2) at follow up (9.1 ± 2.1 h/day; *p* = 0.611). At baseline participants accumulated 4.8 bouts of SB greater than 30 min per day (SD 1.3), this decreased significantly to 4.3 bouts per day (SD 1.6) at follow-up (*p* = 0.010).

### 3.2. Working Day Sedentary Behaviour

Intervention results during the working day are displayed in Table 4. No significant changes between baseline and follow-up were found in any of the outcomes. Participants spent an average of 56.0% (SD 15.2) of their working day sedentary at baseline (4.5 ± 1.5 h/day) and 54.8% (SD 17.4) at follow up (4.4 ± 1.8 h/day; *p* = 0.575). At baseline participants accumulated 2.0 SB bouts greater than 30 min per working day (SD 1.3), and at follow-up this was 1.9 bouts per working day (SD 1.4) at follow-up (*p* = 0.663).

## 4. Discussion

A personalized behavioural intervention aimed at reducing SB and increasing breaks in SB among college employees resulted in a significant decrease in bouts of SB greater than 30 min during the whole day, but not specifically the workday. This is an important finding given the fact that engaging in prolonged periods of unbroken SB is associated with poor health outcomes [31]. The fact that SB bouts were reduced across the whole day, but not specifically the workday is somewhat surprising given that the behavioural intervention was targeted specifically toward SB at work. One possible explanation for this is that participants had more control over their environment and activities outside of work. Therefore, it is possible that the message of the intervention was received, but was harder to put into place within the confines of the working environment. A multi-level intervention, to include environmental restructuring, policy change, and addressing social norms (e.g., walking/standing meetings) may be required to impact on employees’ workplace behaviour. Intervening simultaneously at multiple levels within and across levels and settings is thought to result in greater and longer-lasting behaviour change [32]. For example, a short-term (4-week) multilevel intervention comprising organizational, environmental, and individual change elements to reduce workplace sitting achieved sizeable (>2-h per 8-h workday; −26.5% of workplace time) reductions in workplace sitting [15]. Relatively little is known as to how health promotion programs targeted at the individual behaviour in the worksite might influence workplace norms, the social environment of the workplace and/or workplace policies [33]. However, from an ecological perspective [34] it seems reasonable to assume that a reciprocal relationship might exist. Thus, there is a need for future SB interventions to evaluate program effects at multiple levels.

In the present study, an individually-tailored behavioural intervention did not impact daily sedentary time. This is not entirely unexpected, as the focus of the intervention was breaking up prolonged (>30-min) bouts of SB. Evans et al. reported similar results, with no significant decrease in overall SB but a significant reduction of SB >30 min (1.1 bout/day) in participants who received an educational and email prompts intervention compared to those who only received the educational content [35]. Extending the consultation time, or providing a second consultation, may have allowed for time to focus on decreasing total SB, however we did not want to place a greater burden on participants’ time. The study was also designed to focus on a single behaviour, as participants can feel overwhelmed when asked to change multiple behaviours simultaneously [36].

Previous studies focusing on reducing SB in the workplace have shown conflicting results. A recent systematic review highlighted that studies which implemented environmental changes (e.g., sit-stand desks) noted significant reductions in SB during the working day [37]. However, as mentioned previously, the adoption of these interventions in real life is questionable due to high cost and resources required. When focusing on interventions similar to the present study, which only included educational/behavioural components (e.g., provide information on consequences of behaviour to the individual; goal setting; use prompts/cues), results were inconclusive. However, all interventions showed a reduction in sitting time (pooled reduction of −15.5 min/8-h workday (95% CI: −22.9, −8.2)) which is slightly higher than the mean reduction in the current study which was only about 6 min during the working day. One reason for this could be the relatively low level of SB participants in the current study exhibited. For example, Evans et al. (2012) reported their participants spent 78% of their working day seated compared to 56% in the current sample [35].

Important strengths of this study include the within subject design and the length of time (16 weeks) between the face-to-face intervention and follow-up testing. Also, the detailed objective measurement of multiple features of SB with a validated device designed to differentiate sitting and standing behaviours. The major limitations of this study are the lack of a control group, and a small (*n* = 36) and relatively homogeneous sample. The lack of a suitable control intervention means that our pre-experimental study sheds no light on whether other interventions would result in similar changes. Future controlled trials are warranted which also seek to confirm the current results in larger, more diverse groups. A larger sample size would also present the opportunity to determine whether the observed effects might be moderated by participant characteristics (e.g., age, BMI, occupational role). Finally, it is worth considering that the use of the activPAL device may have resulted in some reactivity (i.e., change in behaviour) of participants due to awareness of being monitored. However, several studies have shown no evidence of reactivity to wearable technology such as accelerometers and pedometers [38,39].

## 5. Conclusions

In this feasibility and pilot study, a personalized SB intervention was successful in reducing number of SB bouts greater than 30 min during the whole day but not the working day. Overall daily sedentary time, number of sitting to upright transitions and number of sedentary bouts less than 30 min were not significantly reduced. These results indicate that consultation based interventions may be effective if goal setting is focused on a specific component of SB (e.g., reducing 30-min bouts, including 10-min active breaks every hour). This study did not include a control group and the results of the study should be confirmed by more structured randomized controlled trials.

## Figures and Tables

**Table 1:** Behavioural Intervention.

Intervention Stage	Objectives	Behaviour Change Techniques	Example
Stage 1	Increase knowledge or understanding of sedentary behaviour (SB) health risks	Information about health consequences and the salience of such consequences from credible sources	Share information on health risks and consequences associated with SB using handout from the American College of Sports Medicine.
Stage 2	Review and reflect upon own SB patterns using activPal output	Develop discrepancy between current behaviour and goal. Consider pros and cons of decreasing SB.	Review current patterns of SB relative to desired SB. List and compare the advantages and disadvantages of sitting less at work.
Stage 3	Develop strategies to decrease SB in the workplace	Action planning	Plan times for standing breaks during the workday.
		Prompts/cues	Keep a set of sneakers or comfortable shoes in the office.
		Habit formation	Stand up every time the phone rings.
		Restructuring the physical and social environment	Purchase or build a sit-stand desk. Promote standing/walking meetings.
Stage 4	Identify barriers to decreasing SB in the workplace and provide solutions	Problem solving	Brainstorm ways to combine work with movement (e.g., walking office hours).
		Prompts/cues	Set electronic reminders to take standing breaks.
		Behaviour substitution	Use a bathroom on a different floor of the building.
Stage 5	Set goal to break up SB bouts > 30 min	Goal setting	Make a behavioural resolution relative to target behaviour on reducing SB bouts >30 min

**Table 2:** Participant baseline data.

Variable	Mean (SD)
Sedentary time (h/day)	9.4 (1.5)
Standing time (h/day)	4.5 (1.3)
Stepping time (h/day)	1.9 (0.5)
Sedentary time (h/working day)	4.5 (1.5)
Standing time (h/working day)	2.5 (1.1)
Stepping time (h/day)	1.0 (0.5)

**Table 3:** Intervention outcomes whole day.

Variable	Baseline	Follow-Up	*p*-Value
Sedentary time (%)	59.1 (8.3)	58.6 (11.2)	0.611
Standing time (%)	28.5 (7.4)	29.0 (9.8)	0.649
Stepping time (%)	12.3 (3.5)	12.5 (4.3)	0.765
Bouts 10–19.99 min	6.8 (2.2)	6.8 (2.2)	0.982
Bouts 20–20.99 min	3.1 (0.9)	3.0 (1.0)	0.917
Bouts > 30 min	4.8 (1.3)	4.3 (1.6)	0.010
Sitting to upright transitions	39.3 (7.8)	37.6 (7.8)	0.175

**Table 4:** Intervention outcomes working day.

Variable	Baseline	Follow-Up	*p*-Value
Sedentary time (%)	56.0 (15.2)	54.8 (17.4)	0.575
Standing time (%)	30.9 (13.3)	32.4 (16.8)	0.428
Stepping time (%)	13.1 (7.1)	12.8 (8.8)	0.748
Bouts 10–19.99 min	3.9 (1.9)	4.0 (2.0)	0.886
Bouts 20–20.99 min	1.6 (0.9)	1.7 (0.9)	0.575
Bouts > 30 min	2.0 (1.3)	1.9 (1.4)	0.663
Sitting to upright transitions	22.3 (8.2)	21.3 (7.7)	0.415
